# A triple drug combination as a novel anti-glioma therapy?

**DOI:** 10.18632/oncotarget.26279

**Published:** 2018-11-06

**Authors:** Yiru Zhang, Georg Karpel-Massler, Markus D. Siegelin

**Affiliations:** Markus D. Siegelin: Department of Pathology & Cell Biology, Columbia University Medical Center, New York, New York, U.S.A.

**Keywords:** triple drug combination therapy, panobinostat, OTX015, sorafenib, glioblastoma

Epigenetic therapies have gained interest amongst the community of scientists and clinicians. Most notably, Histone deacetylase inhibitors (HDACi) and BET bromodomain protein inhibitors (BRD4i) are key players in this field, such as OTX015. Pan-HDACi, such as panobinostat or vorinostat, have received FDA-approval for certain malignancies, e.g. panobinostat is harnessed for the treatment of multiple myeloma, a plasma cell neoplasia that oftentimes clinically manifests with lytic bone lesions and associated pathological fractures of the spine. HDACs are enzymes that remove acetyl groups from histone or non-histone proteins. While modification of histone proteins directly involves the fate of the transcriptome of cells, non-histone modification may have other effects than affecting mRNA production. On a molecular level, these post-translational modifications occur at lysine residues. Enhanced acetylation leads to more accessible chromatin and drives transcription. In this vein, acetylation of histone, H3, at lysine K27 (H3K27ac) is a marker of activation at promoter, enhancer as well as super-enhancer regions and is readily modified by HDACi. Since H3K27ac marks super-enhancer, this histone modification is now widely used in the context of characterizing the super-enhancer landscape of tumors or in the setting of studying the evolvement of drug resistance since drug therapy is known to substantially reprogram the epigenome, including super-enhancers. Consequently, HDACi facilitate the acetylation of H3K27ac. Regarding BRD4, it was found that BRD4 binds to super-enhancers with acetylation marks [1]. Therefore, HDACi facilitate the binding of BRD4 at super-enhancers. In turn, dual targeting of BRD4 and HDAC may potentially result in enhanced synergistic drug efficacy.

**Figure 1:**
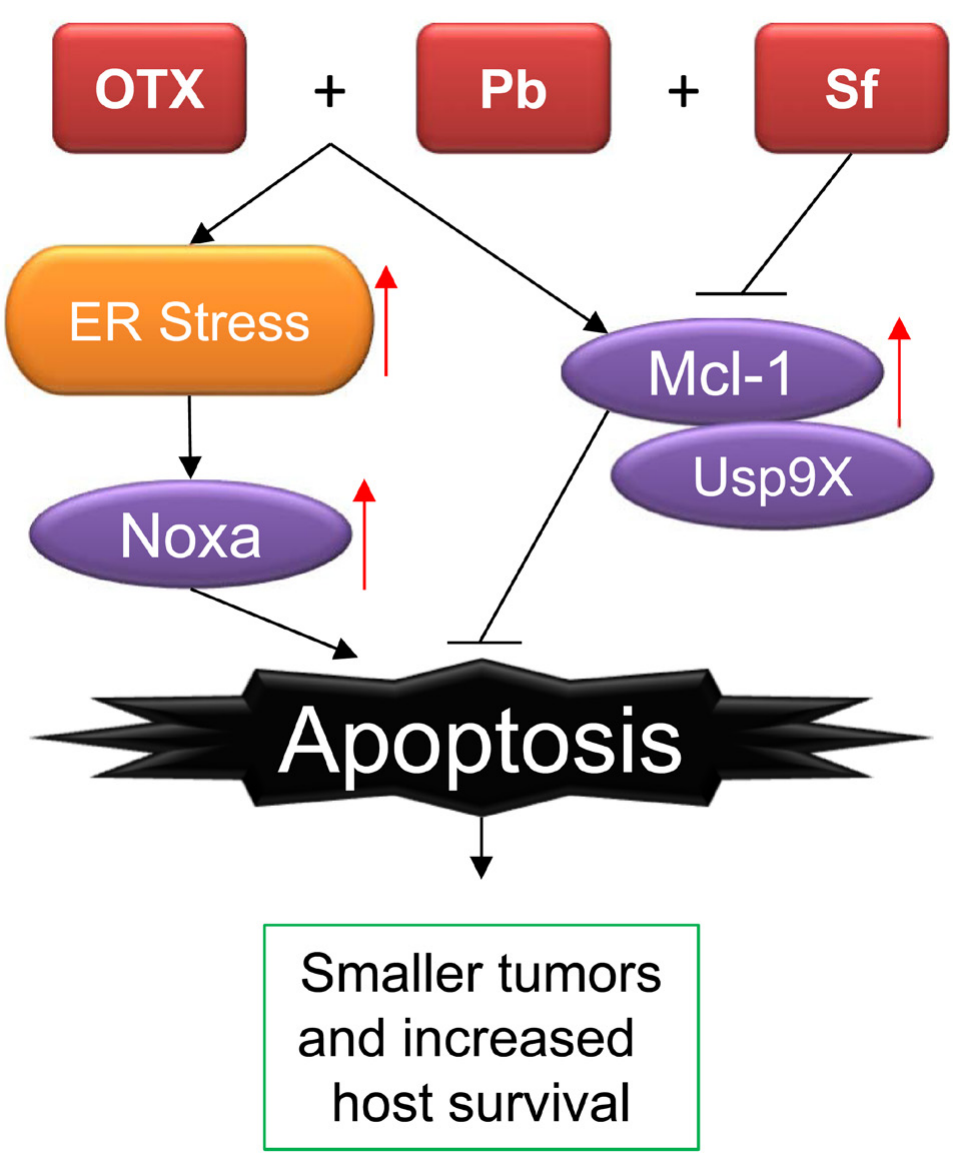
The triple combination therapy elicits enhanced cell death as compared to a dual combination therapy OTX015 and panobinostat upregulate both pro-apoptotic Noxa and Mcl-1. The addition of sorafenib to the combination therapy mitigates OTX015+panobinostat driven increase of Mcl-1 and thereby the triple combination therapy is more potent to induce cell death. Overall, this results in increased animal survival. OTX: OTX015, Pb: Panobinostat, Sf: Sorafenib, ER stress: endoplasmic reticulum stress.

We tested this hypothesis in model systems of glioblastoma since there is a desperate need for more efficient therapies in the light of the fact that the overall survival for patients with glioblastoma remains 12-15 months. Although our article did not provide an in-depth epigenetic analysis of the combination treatment of (OTX015) and HDACi (panobinostat+vorinostat), we found strong synergistic reduction in cellular viability in glioblastoma cells (including stem-like glioblastoma cells which are one of the components that drive glioma recurrence) that was accompanied by energy loss and activation of an endoplasmic reticulum stress response, involving the PERK signaling axis [2] (Figure 1). While the PERK signaling axis was efficient to up-regulate Noxa, we also noted a compensatory increase of anti-apoptotic Mcl-1 by the combination treatment of HDACi and BRD4i, which was not only driven in a transcriptional, but also in a post-translational manner, involving the kinase GSK3β and the deubiquitinase Usp9X that is known to interact with Mcl-1 and promotes its stability. In turn, genetic inhibition of Mcl-1 further enhanced the killing effects of the combination treatment of HDACi+BRD4i, suggesting a potential triple therapy that would antagonize the anti-apoptotic effects elicited by Mcl-1. Very recently, the drug class of BH3-mimetics has received several new members. Amongst those are novel small molecule inhibitors of Mcl-1 that reveal high-affinity to this molecule and have shown efficacy singly or in combination therapies in preclinical animal models with what appears to be an acceptable toxicity profile [3]. In this regard, it is not very surprising that Mcl-1 inhibitors synergize with the more traditional BH3-mimetics, such as ABT263 or even more clinically relevant, ABT199, which has received accelerated FDA approval for certain non-solid malignancies. Some somatic mutations, such as the IDH1 R132H, mutation lower Mcl-1 levels and thereby promote the anti-tumor efficacy of ABT263 [4]. While these earlier BH3-mimetics inhibited only Bcl-2 and Bcl-xL, they lacked inhibition of Mcl-1 and therefore additional means were necessary to target Mcl-1. Instead of utilizing the more novel Mcl-1 selective BH3-mimetics, we have harnessed a clinically validated compound, sorafenib (FDA-approved), to suppress Mcl-1 expression. Sorafenib is a multi-kinase inhibitor and one of its effect is a potent down regulation of Mcl-1 [5]. In line with work done by others, sorafenib silenced the expression of Mcl-1 in our combination therapy, which in turn resulted in more cell death induction. These in vitro findings suggested a potential triple combination therapy, involving panobinostat, OTX015 and sorafenib. We went on to evaluate this triple combination therapy of three clinically validated drugs in several model systems of glioblastoma. We found that the triple combination therapy led to a regression of tumors in the highly aggressive U87-EGFRvIII model system (subcutaneous model system) as well as in a patient derived xenograft (glioblastoma model). Illuminated by these findings, we assessed whether or not the triple combination therapy would extend animal survival in two intracranial murine models. To this purpose, we used two orthotopic models of glioblastoma either derived from a patient-derived xenograft or stem-like glioblastoma cells. Albeit with different efficacies, we found that in both model systems the triple therapy significantly extended overall host survival as compared to vehicle or the combination therapy (panobinostat and OTX015). We believe that these findings herald promise and that this combination therapy might be revisited for a potential clinical trial. Regarding toxicity, we noted no serious side effects after and during the administration of the drugs, albeit that might require more detailed studies. From a broader perspective, it appears that multi-drug combinations might be one venue to target recalcitrant malignancies, such as pancreatic adenocarcinoma or glioblastoma. Inspired in part by the treatment of infectious diseases, multi-drug combinations are an essential part of the treatment for patients with HIV, which has helped to turn this former acute deadly disease into a chronic “condition”. Whether similar successes might be accomplished in brain tumors remains to be seen. However, the results from our study provide at least some hope that a similar success might be possible for treatment resistant cancers.
